# Modified Banxia Xiexin Decoction Ameliorates Polycystic Ovarian Syndrome With Insulin Resistance by Regulating Intestinal Microbiota

**DOI:** 10.3389/fcimb.2022.854796

**Published:** 2022-05-10

**Authors:** Hongyu Zhao, Rufeng Chen, Dongxue Zheng, Feng Xiong, Fan Jia, Jinyuan Liu, Lili Zhang, Nana Zhang, Shiqin Zhu, Yongmei Liu, Linhua Zhao, Xinmin Liu

**Affiliations:** ^1^ Guang’anmen Hospital, China Academy of Chinese Medical Sciences, Beijing, China; ^2^ Graduate College, Beijing University of Chinese Medicine, Beijing, China; ^3^ Institute of Acupuncture and Moxibustion, China Academy of Chinese Medical Sciences, Beijing, China

**Keywords:** polycystic ovary syndrome, insulin resistance, intestinal microbiota, modified Banxia Xiexin Decoction, inflammation, metabolism

## Abstract

**Objective:**

To analyze the characteristics of the intestinal microbiota of polycystic ovarian syndrome with insulin resistance (PCOS-IR) and explore the possible mechanism of modified Banxia Xiexin Decoction in the treatment of PCOS-IR.

**Methods:**

A total of 17 specific pathogen-free (SPF) female Sprague–Dawley (SD) rats, aged 21 days, were selected and randomly divided into the control group (group Z, n = 6), model group (group M, n = 6), and treatment group (group A, n = 5). Letrozole combined with a high-fat diet was used to induce the PCOS-IR model. Rats in group A were treated with modified Banxia Xiexin Decoction for 2 weeks after the end of modeling; then the characteristics of reproductive, metabolic, inflammatory, and intestinal microbiota were compared among three groups.

**Results:**

The PCOS-IR model had an imbalance of intestinal microbiota, and the enriched microbiota was mainly class Coriobacteria, order Clostridiales, and genus *Clostridium_sensu_stricto_1*. Modified Banxia Xiexin Decoction can regulate the disorder of intestinal microbiota diversity, significantly increase the abundance of phyla Verrucomicrobiota Proteobacteria and genera *Akkermansia* and *Blautia*, and decrease the abundance of genus *Clostridium_sensu_stricto_1*.

**Conclusion:**

Genus *Clostridium_sensu_stricto_1* might be the pivotal pathogenic bacteria of PCOS-IR. Modified Banxia Xiexin Decoction may ameliorate PCOS-IR by regulating intestinal microbiota imbalance and improving metabolic disorders.

## Introduction

Polycystic ovary syndrome (PCOS) is one of the common gynecological diseases combined with abnormalities of reproduction and metabolism, which is characterized by ovulation dysfunction, hyperandrogenism, and insulin resistance (IR). It is also the main cause resulting in female anovulatory infertility (Gupta et al., 2019; Cunha and Póvoa, 2021). The incidence rate of PCOS is about 5% to 20% in reproductive-aged women (Barthelmess and Naz, 2014; Ding et al., 2017). PCOS not only affects reproductive function but also increases the risk of long-term complications such as hyperlipidemia, diabetes, metabolic syndrome, cardiovascular disease, and endometrial cancer (Wehr et al., 2011), which seriously endanger women’s physical and mental health (Karjula et al., 2017; Brutocao et al., 2018). IR occurs in about 50%~70% of patients with PCOS (Al-Jefout et al., 2017; Dahan et al., 2019), and although it is not one of the diagnostic criteria of PCOS, it plays an important role in the pathogenesis of PCOS (He and Li, 2020). IR is not merely an important pathophysiological change but also a vital hub connecting reproductive abnormalities and metabolic disorders of PCOS (Carreau and Baillargeon, 2015). IR and its resulting compensatory hyperinsulinism are linked to hyperandrogenemia in various ways; for instance, they stimulate the pituitary to secrete luteinizing hormone (LH) (Teede et al., 2007; Szeliga et al., 2022) and decrease hepatic production of sex hormone-binding globulin (SHBG) (Calcaterra et al., 2021). It can also affect the metabolic function of the body by affecting the level of blood lipids and a variety of adipocytokines (Teede et al., 2007; Zheng et al., 2017; Calcaterra et al., 2021). Therefore, taking IR as the starting point to research PCOS has great significance.

In recent years, a number of studies have shown that the intestinal microbiota, also known as the “second genome of the human body,” may play an important role in the pathogenesis of polycystic ovarian syndrome with IR (PCOS-IR) (Thackray, 2019; Starling, 2021). Tremellen et al. firstly proposed the hypothesis of “gut barrier-endotoxemia-inflammation”, considering that changes in dietary conditions can lead to intestinal microbiota disorders, impaired gut mucosal barrier, and increased intestinal mucosal permeability, which allow the produced “lipopolysaccharide (LPS)” to enter the bloodstream and then cause IR and PCOS (Vrieze et al., 2010; Tremellen and Pearce, 2012). The disturbance of the microbiota can induce abnormal glucose metabolism, hyperandrogenemia, and abnormal follicular development in PCOS (Xie et al., 2016; Qi et al., 2019; Han et al., 2021), and a healthy gut microbiota protects against reproductive and metabolic dysregulation (Torres et al., 2019; Liyanage et al., 2021).

Modern research shows that Banxia Xiexin Decoction derived from “Typhoid Theory” can regulate intestinal microbiota disorder, reduce blood glucose, and improve IR (Yang et al., 2019; Yang et al., 2021). Preliminary clinical studies of our research group have shown that Banxia Xiexin Decoction is an effective prescription for the treatment of PCOS, which can enhance insulin sensitivity, correct glucose metabolism disorders, and promote the recovery of ovulation and spontaneous menstruation (Liu et al., 2016). Therefore, this study used a high-fat diet combined with letrozole to induce PCOS-IR model rats, then intervened with modified Banxia Xiexin Decoction, and analyzed the changes in the reproductive hormone, glucose metabolism, inflammatory factors, and intestinal microbiota. This study could help to better understand the potential mechanisms of modified Banxia Xiexin Decoction in the therapy of PCOS-IR from the perspective of gut microbiota.

## Methods

### Animals

Twenty-one-day-old female Sprague–Dawley (SD) rats (specific pathogen-free (SPF) grade) were raised in the Animal Experiment Center of Guang’anmen Hospital, Chinese Academy of Chinese Medical Sciences, and their experimental animal license number is SCXK (Beijing) 2019-0008. The feeding conditions were as follows: room temperature, 22°C ± 2°C; relative humidity, 50% ± 10%; and 12-h light/12-h darkness, alternating cycles. All rats drank and ate freely. There were two kinds of feed, namely, ordinary feed (energy supply ratio: protein 22.47%, fat 12.11%, and carbohydrate 65.42%) and high-fat feed (energy supply ratio: protein 19.5%, fat 23.8%, and carbohydrate 56.7%); the main ingredients are casein, starch, maltodextrin, sucrose, fiber vegetarian, soybean oil, etc. The rats and feed were provided by Beijing Huafukang Biotechnology Co., Ltd. This experiment was reviewed by the Experimental Animal Ethics Committee of the China Academy of Chinese Medicine (Approval Number: IACUC-GAMH-2017-002).

### Herbal Materials

Modified Banxia Xiexin Decoction (ingredients: Rhizoma Pinelliae (Qing Banxia) 9 g, Scutellaria (Huanqin) 20 g, Rhizoma Coptidis (Huanlian) 10 g, Rhizoma Zingiberis (Ganjiang) 9 g, Codonopsis pilosula (Dangshen) 12 g, Licorice (Gancao) 12 g, Fructus Ziziphi Jujubae (Dazao) 9 g, Epimedium (Xianlingpi) 15 g, and Fruit of Chinese wolfberry (Gouqizi) 30 g. All Chinese herbs were supplied by the pharmacy of Guang’anmen Hospital, Chinese Academy of Chinese Medical Sciences, and then made into Banxia Xiexin Tang extract (3.9 g of crude drug per ml of extract); the extract was stored in a refrigerator at 4°C for later use.

### Establishment of Polycystic Ovarian Syndrome With Insulin Resistance Model

Seventeen rats were randomly allocated to 3 groups: control group (group Z, n = 6), model group (group M, n = 6), and treatment group (group A, n = 5). To induce the PCOS-IR model, rats in the model group and treatment group were gavaged with 2 ml LET-CMC solution (1 mg/kg/day of letrozole dissolved in 1% carboxymethyl cellulose), while the control group was gavaged with 2 ml of normal saline, lasting 3 weeks. Then group A was given modified Banxia Xiexin Decoction (26.46 g/kg/day) for 2 weeks; at the same time, two other groups were given the same volume of normal saline. The dose was calculated according to the “equivalent dose conversion of body surface area,” (Xu et al., 2002) and it is administered at 2 times the clinically equivalent dose.

### Serum Sample Collection and Analysis

The morning after 2 weeks of treatment, blood samples were collected from the abdominal aorta of anesthetized rats using a procoagulation tube and centrifuged at 3,000 rpm for 20 min using the 4°C centrifuge (ICT15RE, Hitachi, Tokyo, Japan). Then the upper supernatants were carefully transferred to the EP tube using a pipette gun and immediately stored at a −80°C refrigerator for further detection. The levels of estradiol (E2, JEB-13691, Jinyibo, Wuxi, China), testosterone (T, ab10866, Abcam, Cambridge, MA, USA), LH (JEB-13706, Jinyibo, China), follicle-stimulating hormone (FSH; JEB-13680, Jinyibo, China), fasting insulin (FINS, ab100578, Abcam, USA), LPS (JEB-13984, Jinyibo, China), and tumor necrosis factor-α (TNF-α, ERC102a.96, Xinbosheng, China) were analyzed by ELISA kits, which were operated in strict accordance with each instruction manual. Blood glucose (Glu) was measured using Roche glucometer before anesthesia. The homeostasis model assessment of IR index (HOMA-IR) and HOMA of beta-cell function index (HOMA-beta) was calculated using the following formulas:


HOMA−IR = Glu (mmol/L)×FINS(mIU/L)/22.5.

HOMA−beta=20×FINS (mIU/L)/[FPG (mM)−3.5] × 100%.

### Ovarian Tissue Collection and Observation

Rats were euthanized by cervical dislocation after anesthesia. The left ovaries of rats were collected, then fixed with 4% paraformaldehyde solution, embedded in conventional paraffin (EG140H, Leica, Wetzlar, Germany), sectioned at a thickness of 5 μm (RM2135, Leica), and processed according to the standard procedure of H&E staining, and ovary morphological changes were observed using a light microscope (Olympus, Tokyo, Japan).

### Fecal Sample Collection

Fecal samples were collected by rectal pressing method before the rats were anesthetized; 3–5 fecal samples were collected from each rat and immediately stored at −80°C.

### DNA Extraction and PCR Amplification

Microbial community genomic DNA was extracted from rat fecal samples using the E.Z.N.A.^®^ soil DNA Kit (Omega Bio-Tek, Norcross, GA, USA) according to the manufacturer’s instructions. The DNA extract was checked on 1% agarose gel, and DNA concentration and purity were determined with NanoDrop 2000 UV-vis spectrophotometer (Thermo Scientific, Wilmington, DE, USA). The hypervariable region V3–V4 of the bacterial 16S rRNA gene was amplified with primer pairs 338F (5′-ACTCCTACGGGAGGCAGCAG-3′) and 806R (5′-GGACTACHVGGGTWTCTAAT-3′) by an ABI GeneAmp^®^ 9700 PCR thermocycler (ABI, Los Angeles, CA, USA). The PCR amplification of 16S rRNA gene was performed, as follows: initial denaturation at 95°C for 3 min, followed by 27 cycles of denaturing at 95°C for 30 s, annealing at 55°C for 30 s, extension at 72°C for 45 s, single extension at 72°C for 10 min, and end at 4°C.

### Illumina MiSeq Sequencing and Processing of Data

Purified amplicons were pooled in equimolar and paired-end sequenced on an Illumina MiSeq PE300 platform (Illumina, San Diego, CA, USA) according to the standard protocols by Majorbio Bio-Pharm Technology Co. Ltd. (Shanghai, China). The raw 16S rRNA gene sequencing reads were demultiplexed, quality-filtered by fastp version 0.20.0 (Chen et al., 2018), and merged by FLASH version 1.2.7 (Magoč and Salzberg, 2011). Operational taxonomic units (OTUs) were clustered by UPARSE version 7.1 with ≥97% similarity (Edgar, 2013), and chimeric sequences were identified and removed. The taxonomy of each OTU representative sequence was analyzed by RDP Classifier version 2.2 (Wang et al., 2007) against the 16S rRNA database (silva138/16s_bacteria) using a confidence threshold of 0.7. In order to observe the α-diversity of the intestinal microbial community, including richness and diversity, the number of Ace and Shannon indexes was calculated by the built-in commands of Mothur (https://www.mothur.org/wiki/Download_mothur). Principal coordinate analysis (PCoA), based on weighted UniFrac distance, and partial least squares discriminant analysis (PLS-DA) were used to reflect the β-diversity of intestinal microbiota, which mainly express the significance of intestinal microbiota difference among groups. To obtain significantly different bacterial species among groups, linear discriminant analysis (LDA) effect size (LEfSe) was applied. Here, LDA values >4.0 were set as the thresholds for biomarker identification. Meanwhile, in order to predict the functional profile of the intestinal microbiota community, the Phylogenetic Investigation of Communities by Reconstruction of Unobserved States (PICRUSt, http://picrust.github.io/picrust/) was applied to perform microbiota functional prediction. After OTU sequences were clustered according to 97% similarity, the OTU abundance was standardized by PICRUSt, which meant that the influence of copy number of 16S marker gene in species genome was removed, and the information of Clusters of Orthologous Groups of proteins (COG) family and Kyoto Encyclopedia of Genes and Genomes (KEGG) corresponding to each OTU was obtained through the Greengene ID (http://greengenes.secondgenome.com/) and then according to the different databases, i.e., EggNOG (evolutionary genealogy of genes: Non-supervised Orthologous Groups, http://eggnog.embl.de/) and KEGG (http://www.genome.jp/kegg/); the COG function and each hierarchy level of KEGG pathways could be obtained.

### Data Statistical Analysis

SPSS statistical package 22.0 and GraphPad Prism version 8.3.0 were used for the statistical analysis of clinical data, and all data were expressed as means ± SD or as medians with interquartile ranges. The Kolmogorov–Smirnov test of normality was applied to all data. One-way ANOVA was used to evaluate the statistical significance of differences for normally distributed data. Then homogeneity of variances was tested, and if the variances were homogeneous, Scheffe’s test was used; if not, Dunnett’s method was used. For non-normally distributed data, the Mann–Whitney U-test (two-group comparison) and the Kruskal–Wallis test (more than two groups) can be used.

For bioinformatics analysis, Wilcoxon rank-sum test was used to analyze the differences in α-diversity index (including Ace and Shannon indexes) and the abundance of intestinal microbiota community between two groups. To analyze the significant difference in intestinal microbiota community (i.e., β-diversity) among groups, analysis of similarity (ANOSIM), based on weighted UniFrac distance, was performed with 999 iterations. *p* < 0.05 was considered statistically significant.

## Results

### General Characteristics of the Experimental Rats

**Figure 1:**
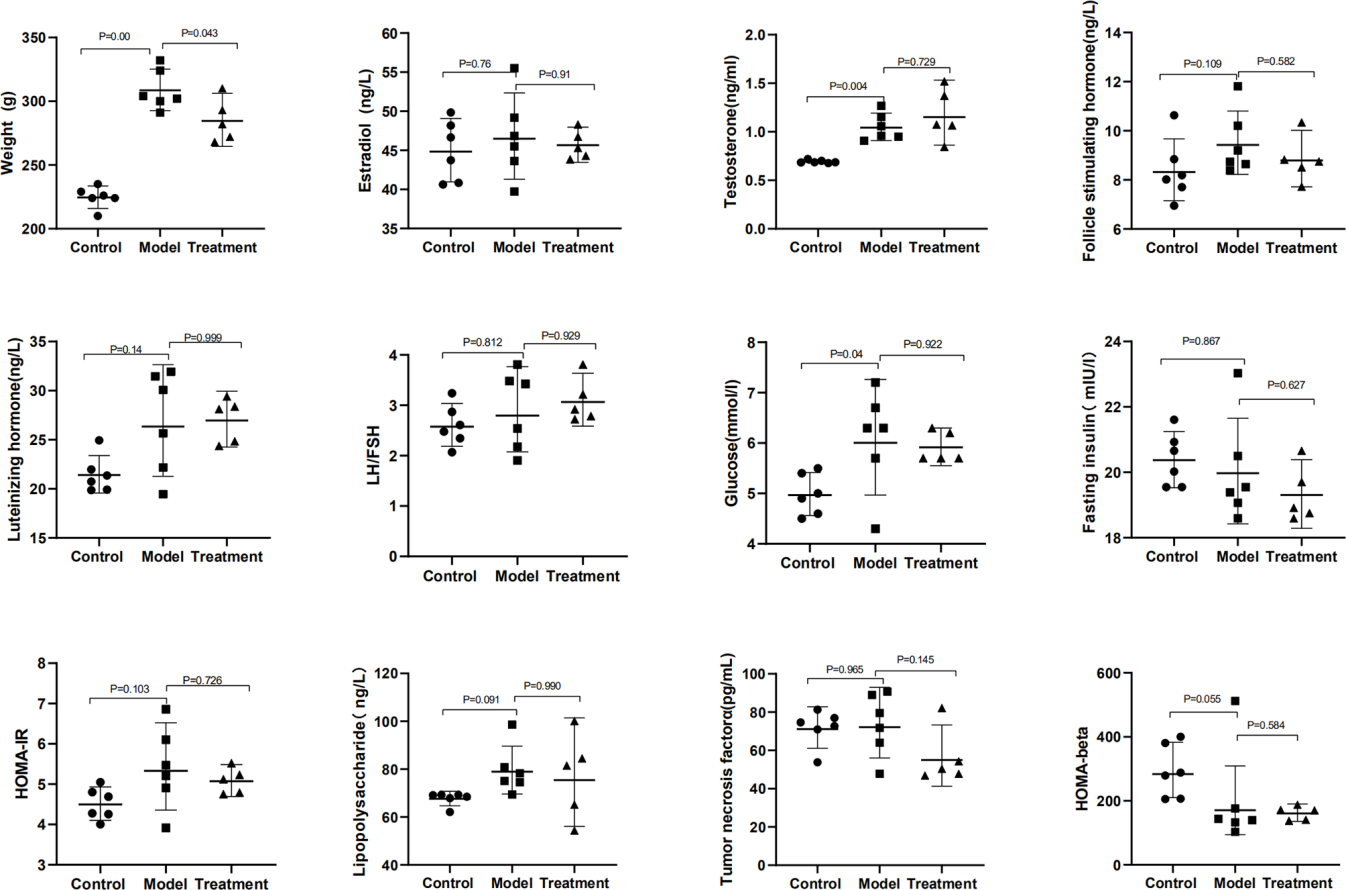
Characteristics of reproductive hormones, glucose metabolism, and inflammatory factors.

The general characteristics of all rats, including body weight, female hormones, and related indicators of glucose metabolism, are summarized in **Table 1** and **Figure 1**. Compared with those in the control group, the body weight, serum T, and Glu significantly increased in the model group (*p* = 0.000, 0.004, and 0.04); FSH, LH, HOMA-IR, and LPS had an increasing trend (*p* > 0.05); HOMA-beta had a downward trend (*p* > 0.05), showing the change of endocrine and metabolic disorders. Compared with those in the model group, the body weight in the treatment group significantly decreased (*p* = 0.043), and the FSH, HOMA-IR, and TNF-α had a downward trend (*p* > 0.05). There was no significant difference in E2, LH/FSH, and FINS among these groups (*p* > 0.05).

**Table 1:** Changes in reproductive, metabolic, and inflammatory factors in all rats.

Parameters	Control (n = 6)	Model (n = 6)	Treatment (n = 5)
Weight (g)	224.67 ± 8.29	308.83 ± 15.70^**^	285.00 ± 17.00^#^
E2 (ng/L)	44.98 ± 3.85	46.74 ± 5.35	45.70 ± 1.83
T (ng/ml)	0.69 ± 0.02	1.05 ± 0.14^**^	1.17 ± 0.27
FSH (ng/L)	8.10 (7.51, 9.29)	8.96 (8.58, 10.61)	8.75 (8.12, 9.58)
LH (ng/L)	21.47 ± 1.89	26.79 ± 5.20	27.03 ± 2.26
LH/FSH	2.60 ± 0.41	2.89 ± 0.78	3.09 ± 0.44
Glu (mmol/l)	4.98 ± 0.41	6.08 ± 1.00^*^	5.92 ± 0.30
FINS (mIU/l)	20.38 ± 0.83	20.02 ± 1.60	19.32 ± 0.86
HOMA-IR	4.51 ± 0.40	5.71 ± 0.78	5.08 ± 0.32
HOMA-beta	283.66 (206.35, 385.51)	141.79 (125.38, 260.29)	170.47 (139.24, 179.85)
LPS (ng/L)	67.71 ± 2.80	79.48 ± 10.14	71.39 ± 14.17
TNF-α (pg/ml)	71.71 ± 9.50	79.03 ± 11.31	56.28 ± 14.73

Data are presented as mean ± SD or as medians with interquartile ranges.

E2, estradiol; T, testosterone; FSH, follicle-stimulating hormone; LH, luteinizing hormone; LH/FSH, the ratio of LH to FSH; Glu, fasting plasma glucose; FINS, fasting plasma insulin; HOMA-IR, homeostasis model assessment of insulin resistance; HOMA-beta, homeostasis model assessment for beta-cell function; LPS, lipopolysaccharide; TNF-α, tumor necrosis factor-α.

^*^p < 0.05, ^**^p < 0.01 for control vs. model group.

^#^p < 0.05 for model vs. treatment group.

### Ovarian Histomorphological Changes

**Figure 2:**
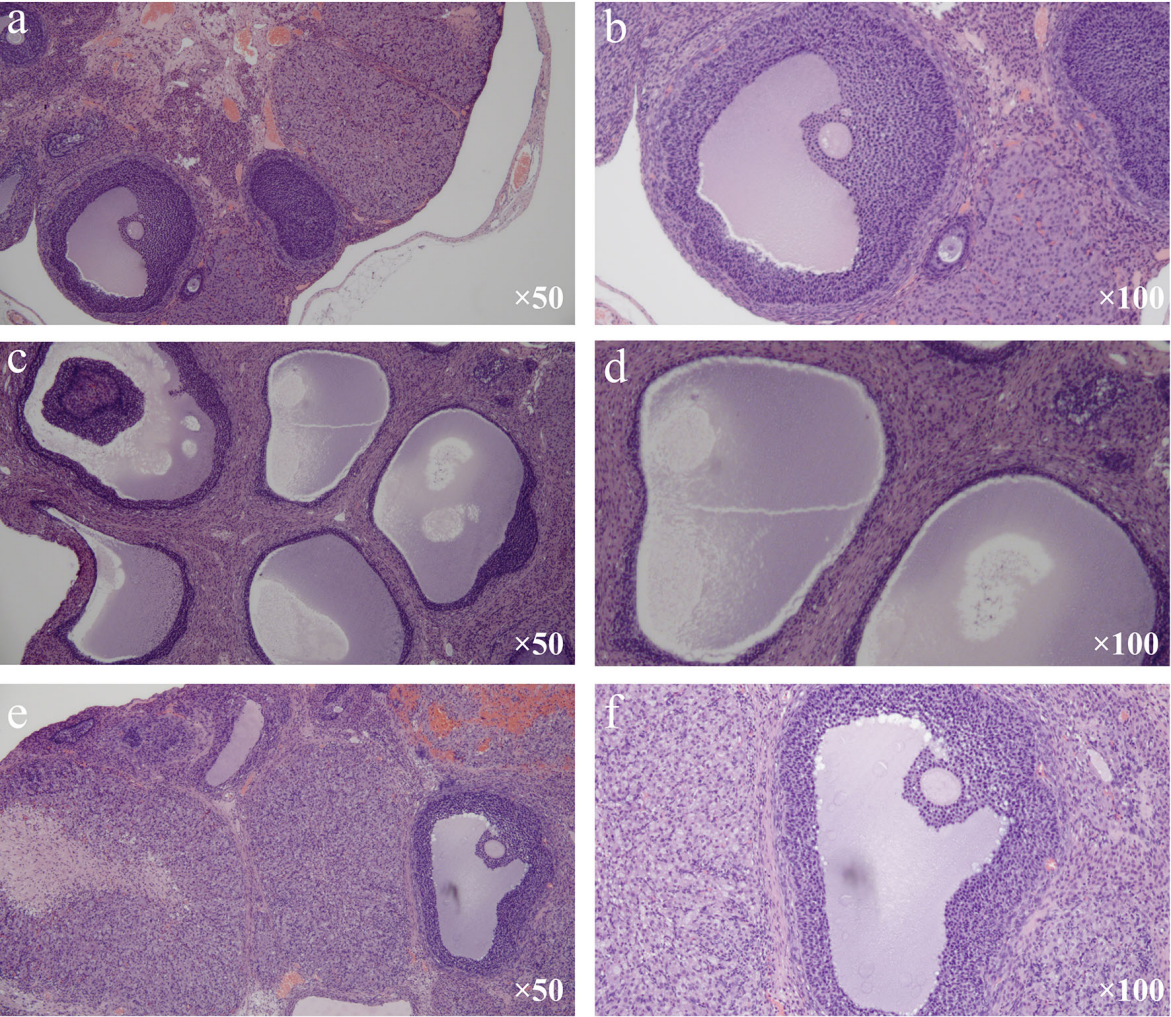
Changes in ovarian histomorphological (H&E stain). **(A, B)** Control group. **(C, D)** Model group. **(E, F)** Treatment group.

Under the light microscope, follicles in different developmental stages, including a mature follicle, and many corpora lutea were observed in the control group. Meanwhile, in the mature follicle, oocytes could be seen, and granulosa cells were arranged neatly and had multiple layers (**Figures 2A, B**). In the model group, many cystic dilated follicles with irregular structures can be seen, and the granulosa cell layer was reduced and even disappeared. Mature follicle and corpora lutea can hardly be seen (**Figures 2C, D**). The morphology of ovarian tissue in the treatment group was similar to that in the control group. A mature follicle and corpora lutea could be observed in the treatment group, and the layers of granulosa cells in follicles were more than those in the model group (**Figures 2E, F**).

### α- and β-Diversity of Intestinal Microbiota Community

**Figure 3:**
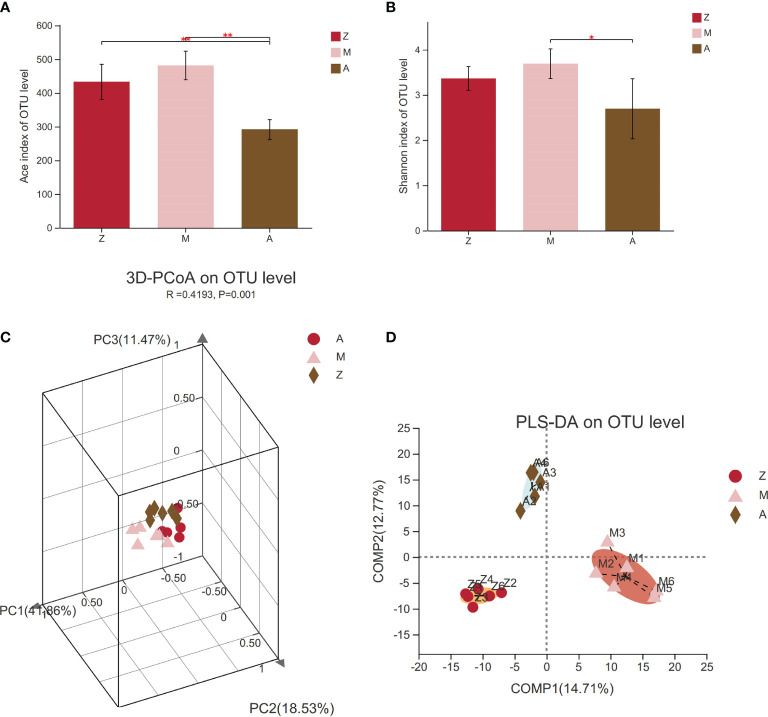
Diversity of intestinal microbiota community. Z, control group; M, model group; A, treatment group. PCoA, principal coordinate analysis; PLS-DA, partial least squares discriminant analysis. **(A)** Ace index. **(B)** Shannon index. **(C)** PCoA. **(D)** PLS-DA.*p < 0.05; **p < 0.01.

α-Diversity index analysis can reflect the richness and diversity of intestinal microbiota communities in a specific regional environment; the greater the value, the higher the community diversity. The results are shown in the box diagram (**Figures 3A, B**). Compared with those in the control group, the Ace and Shannon indexes in the model group had an increasing trend (*p* > 0.05); meanwhile, the Ace index and Shannon index in the treatment group decreased significantly (*p* < 0.01, *p* < 0.05), which indicated that modified Banxia Xiexin Decoction can regulate the α-diversity of PCOS-IR rats.

The β-diversity of the intestinal microbiota was expressed by PCoA and PLS-DA. The PCoA results showed that the intestinal microbiota was significantly different among the three groups (R = 0.4193, *p* = 0.001, **Figure 3C**). From PLS-DA, we can clearly see the distance of samples between groups. The greater the distance, the bigger the difference. Distances between groups meant that the samples in the model group can be clearly distinguished from other groups, while the samples in the treatment group can also be completely discriminated from the control group (**Figure 3D**).

### Analysis of Intestinal Microbiota Community

**Figure 4:**
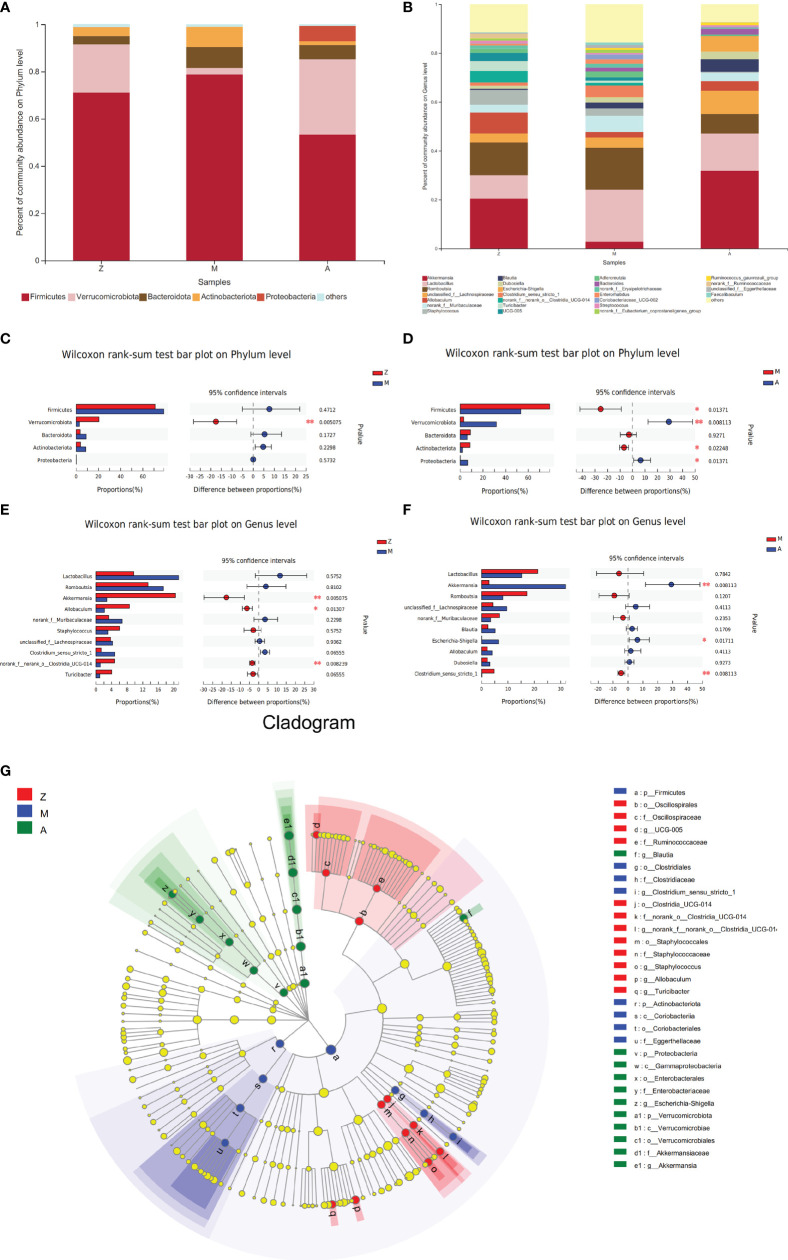
Composition and difference analysis of intestinal microbiota at phylum and genus levels. Z, control group; M, model group; A, treatment group. **(A)** Percent of community abundance on Phylum level. **(B)** Percent of community abundance on Genus level. **(C)** The microbiota proportions’ difference between Group Z and M on Phylum level. **(D)** The microbiota proportions’ difference between Group M and A on Phylum level. **(E)** The microbiota proportions’ difference between Group Z and M on Genus level. **(F)** The microbiota proportions’ difference between Group M and A on Genus level. **(G)** Cladogram by Lefse analysis. *p < 0.05; **p < 0.01.

On the phylum level, 5 phyla were identified, arranged in descending order of relative abundance, namely, Firmicutes, Verrucomicrobiota, Bacteroidota, Actinobacteriota, and Proteobacteria (**Figure 4A**). Compared with that in the control group, Verrucomicrobia in the model group showed a significant decrease (*p* = 0.005, **Figure 4C**). Compared with those in the model group, Firmicutes and Actinobacteriota showed a significant decrease (*p* = 0.014 and 0.022), and the abundance of Verrucomicrobiota and Proteobacteria was significantly increased in the treatment group (*p* = 0.008 and 0.013, **Figure 4D**).

On the genus level, identified bacterial taxa are represented in **Figure 4B**. The abundance of genera *Akkermansia*, *Allobaculum*, and *norank_f:norank_o:Clostridia_UCG-014* in the model group was significantly less than that in the control group (*p* = 0.005, 0.013, and 0.008, **Figure 4E**). Compared with those in the model group, genera *Akkermansia* and *Escherichia-Shigella* were significantly increased in the treatment group (*p* = 0.008 and 0.017, **Figure 4F**), while *Clostridium_sensu_stricto_1* showed an obvious decrease (*p* = 0.008, **Figure 4F**).

Next, we used LEfSe to evaluate significant differences in the relative abundance of bacterial orders, classes, and genera among the three groups. In the control group, order Clostridia_UCG-014, order Staphylococcales, order Oscillospirales, and genus *Allobaculum* presented significant enrichment. The PCOS-IR model group was enriched in genus *Clostridium_sensu_stricto_1* (order Clostridiales) and order Coriobacteriales (class Coriobacteriia). In the treatment group, the abundance of genera *Akkermansia* (phylum Verrucomicrobiota), *Escherichia-Shigella* (phylum Proteobacteria), and *Blautia* was more plentiful (LDA score >4.0, *p* < 0.05, **Figure 4G**).

### Predictive Functional Profiling of Microbiota Community

**Figure 5:**
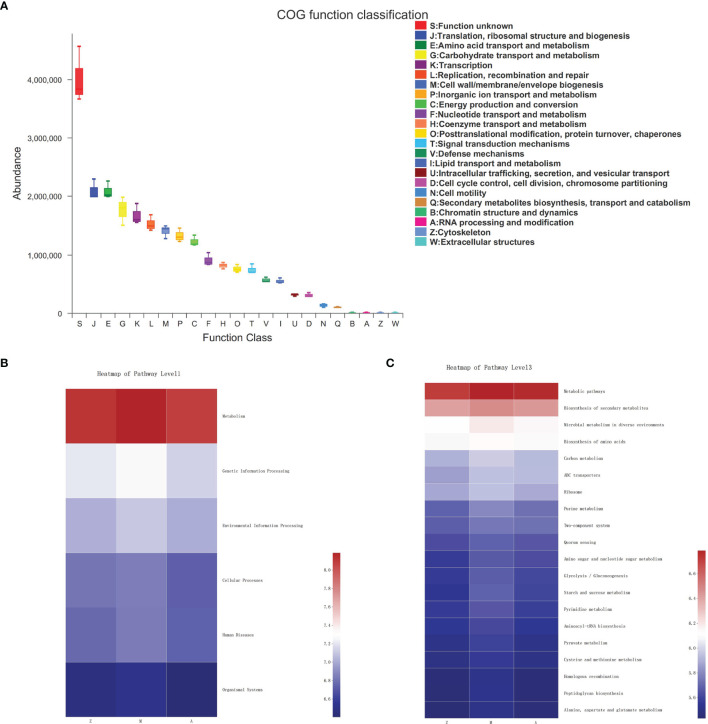
Predictive functional profiling of microbiota community. Z, control group; M, model group; A, treatment group. **(A)** COG function classification. **(B)** KEGG Pathway Level 1. **(C)** KEGG Pathway Level 3.

In the fecal samples of three groups, the COG function of gut microbiota was predicted including translation, ribosomal structure, and biogenesis; amino acid transport and metabolism; carbohydrate transport and metabolism; transcription; replication, recombination, and repair; cell wall/membrane/envelope biogenesis; inorganic ion transport and metabolism; and energy production and conversion (**Figure 5A**). At KEGG level 1, metabolism accounted for a large proportion of pathway prediction, followed by environmental information processing, genetic information processing, cellular processes, etc. (**Figure 5B**). At KEGG level 3, the predicted results indicated that the microbiota may be mainly involved in functions such as metabolic pathways, biosynthesis of secondary metabolites, microbial metabolism in diverse environments, biosynthesis of amino acids, and carbon metabolism to exert a therapeutic effect (**Figure 5C**).

## Discussion

As a hot and difficult point in the research of gynecological endocrine diseases, PCOS-IR has the characteristics of elevated T and LH/FSH (Le et al., 2019), and it is often associated with abnormal glucose and lipid metabolism. Therefore, both reproduction and metabolism need to be taken into account in the treatment of PCOS, which has great significance for improving women’s reproductive health and preventing long-term complications. This study showed that the modified Banxia Xiexin Decoction significantly reduced body weight and promoted follicular development and maturation. However, the regulation of reproductive hormones was not obvious.

The intestinal microbiota is composed of a large amount of parasitic microorganisms in the human intestinal tract, which plays an important role in host physiology, such as immunity, metabolism, protection of intestinal epithelial barrier, and production of short-chain fatty acids (Kelly et al., 2015; Wahlstrom et al., 2016; Barko et al., 2018). Despite this, when the internal and external environments of the body change, such as the changes in diet, disease, or misused antibiotics, it will cause an imbalance of gut microbiota, which is widely recognized as malnutrition of intestinal microbiota (Arroyo et al., 2019; Torres et al., 2019; Chu et al., 2020; Zhou et al., 2020). In recent years, numerous studies have shown that there is a relationship between microbiota imbalance and PCOS (Rizk and Thackray, 2020; Parker et al., 2022). The imbalance of intestinal microbiota can cause LPS to enter the bloodstream by increasing intestinal permeability (Tremellen and Pearce, 2012; Chang et al., 2021). LPS is considered to have the effect of endotoxin, which can activate CD14/TLR4 receptors on the surface of immune cells and induce inflammatory response (Festi et al., 2014; Hersoug et al., 2016; Nighot et al., 2019; Chang et al., 2021). Chronic low-grade inflammation is considered to be a key factor in the pathogenesis of PCOS (Vrieze et al., 2010; Tremellen and Pearce, 2012; Crommen and Simon, 2017; Patel, 2018; Thackray, 2019; Abraham Gnanadass et al., 2021; He et al., 2021). Chang et al. (2021) found that there was a disorder of intestinal microbiota in the PCOS rat model, as well as an abnormal state of inflammatory factors, such as LPS, TNF-α, and IL-6. There was no significant difference in inflammatory factors in this study, which may be related to the small sample sizes. However, judging from the trend of increasing inflammatory factors in the model group and decreasing inflammatory factors in the treatment group, it could be inferred that modified Banxia Xiexin Decoction has a potential regulatory effect on inflammation disorders.

From the comparison of microbiota, genus *Akkermansia* significantly decreased in the model group and increased after treatment, indicating that *Akkermansia* may be a promising probiotic for treating PCOS-IR. Previous studies have shown that *Akkermansia* can reduce the body weight and total cholesterol of obese patients, increase insulin sensitivity, improve IR, and reduce inflammatory reaction (Shin et al., 2014; Dao et al., 2016; Depommier et al., 2019), and its mechanism may be adjusting the thickness of intestinal mucosa and maintaining the integrity of intestinal barrier (Derrien et al., 2004). As a potential probiotic, *Blautia* was significantly enriched in the treatment group, and it was proved to play certain roles in metabolic diseases, inflammatory diseases, and biotransformation (Eren et al., 2015; Liu et al., 2021; Zheng et al., 2021). Meanwhile, *Clostridium_sensu_stricto_1*, which was thought to cause inflammation and lead to severe intestinal infections (Fletcher et al., 2021), was significantly higher in the model group and significantly lower in the treatment group. These results suggested that *Clostridium_sensu_stricto_1* may be one of the key pathogens causing PCOS-IR, and modified Banxia Xiexin Decoction can promote the growth of probiotics and inhibit the proliferation of harmful bacteria. It also could be observed that *Allobaculum* had a decreasing trend and *norank_f_Muribaculaceae* bacteria had an increasing trend in the model group while showing opposite changes after treatment. Although the specific functions of these two bacteria are still unclear, studies have reported that Muribaculaceae was linked to obesity and had an increasing abundance in obese mice (Chevalier et al., 2020; Liu et al., 2021; Yu et al., 2021), and *Allobaculum* was inversely proportional to fat quality (Cox et al., 2014), suggesting that they may be related to lipid metabolism.

Functional prediction analysis indicated that the intestinal microbiota might play an important role in affecting metabolic functions, which was consistent with previous studies (Vojinovic et al., 2019; Pan et al., 2021). Microbiota is mainly involved in a variety of metabolic pathways, such as bile acids (Parséus et al., 2017; Pathak et al., 2018), short-chain fatty acids (Abdul Rahim et al., 2019 ; Sanna et al., 2019), amino acids, methylamine, and indoles (Abdul Rahim et al., 2019). Vojinovic et al. (2019) found that the abundance of some bacterial groups was negatively correlated with low-density lipoprotein, triglycerides, and fatty acids. Aoki et al. (2017) found that mice given *Bifidobacterium* have reduced content of visceral fat and subcutaneous fat and reduced blood glucose level. In our study, modified Banxia Xiexin Decoction can potentially improve the sensitivity of insulin and correct the disorder of glucose metabolism to varying degrees, showing that intestinal microbiota may improve glucose metabolism to achieve the purpose of trp>

More interestingly, the tendency of α-diversity increased in the model group, contrary to the majority of studies that believed that the α-diversity decreased in PCOS patients/animals (Kelley et al., 2016; Insenser et al., 2018; Torres et al., 2018; Jobira et al., 2020). However, there were still a small number of studies showing that α-diversity in patients with PCOS increased (Zhu et al., 2020; Yang et al., 2021). Yang et al. (2021) found that the diversity of bacterial microbiota increased significantly in PCOS patients and animal models; Zhu et al. (2020) also found that α-diversity in PCOS-IR model rats had an increasing trend. As we all know, diet is one of the most important environmental factors that change the structure of the microbiota (David et al., 2014; Zheng et al., 2021). Whether the increase in α-diversity was caused by a high-fat diet or a rising proportion of harmful bacteria needs further study.

*Escherichia-Shigella* is generally considered a harmful bacterium that can cause multiple infections (Croxen et al., 2013). Studies had shown that obese patients or rats were prone to abnormal elevation of *Escherichia-Shigella* after Roux-en-Y gastric bypass (Kong et al., 2013; Dang et al., 2021), and they believed that this may be related to multiple conjugated bile acids (Dang et al., 2021). Whether the increase of *Escherichia-Shigella* observed in this study is associated with the metabolic changes of bile acids or something else needs further research.

In the present study, it can be clearly seen that the microbiota disorders had been significantly regulated, follicular growth had been promoted, and the improvement of glucose metabolism characteristics and inflammatory factors had only a changing trend. The small sample sizes may be responsible for this. Another limitation of this study is that we did not have a separate control group with a high-fat diet, which cannot make a strong explanation for the increasing α-diversity. The last is that after we screened out the microbiota with significant differences among groups, such as genera *Akkermansia* and *Clostridium_sensu_stricto_1*, we did not conduct further experiments, such as transplantation into germ-free rats, to verify the exact role of these bacteria in PCOS-IR. Of course, we will gradually solve these problems in the following research.

In conclusion, the current study demonstrated that the intestinal microbiota in the PCOS-IR model was significantly out of balance. *Clostridium_sensu_stricto_1* may be the key pathogen of PCOS-IR. The effect of modified Banxia Xiexin Decoction ameliorating PCOS-IR may be achieved by adjusting the disorder of intestinal microbiota and then improving the metabolic disorder of the body, and *Akkermansia* may play an important role in the treatment.

## Data Availability Statement

The data presented in the study are deposited in the National Center for Biotechnology Information(NCBI) BioProject repository, accession number PRJNA823038.

## Ethics Statement

The animal study was reviewed and approved by the Ethics Committee of Experimental Animals, the China Academy of Chinese Medicine.

## Author Contributions

HZ took in charge of the whole process of the experiments, and the writing and modification of this paper. RC participated in the experiments and revised this manuscript. DZ participated in the experiments and guided the writing. FX participated in the translation and modification of this paper and sorted all pictures. FJ, JL and LLZ participated in the experimental processes. SZ and NZ were responsible for parts of the translation of the article. YL guided the experimental methods. LHZ directed the design of the project. XL coordinated and conducted all the work. All authors listed had made a substantial, direct, and intellectual contribution to the work and approved it for publication. 

## Funding

This work was supported by the National Natural Science Foundation of China (81674011).

## Conflict of Interest

The authors declare that the research was conducted in the absence of any commercial or financial relationships that could be construed as a potential conflict of interest.

## Publisher’s Note

All claims expressed in this article are solely those of the authors and do not necessarily represent those of their affiliated organizations, or those of the publisher, the editors and the reviewers. Any product that may be evaluated in this article, or claim that may be made by its manufacturer, is not guaranteed or endorsed by the publisher.
